# A gaint mesenteric cystic lymphangioma of small intestinal in an adult: A case report and literature review

**DOI:** 10.1097/MD.0000000000042394

**Published:** 2025-05-23

**Authors:** Wenxian Yin, Ruyi Yu, Dong Xia

**Affiliations:** a Department of Clinical Medicine, Southwest Medical University, Luzhou, Sichuan Province, China; b Department of General Surgery, Gastrointestinal Group, Affiliated Hospital of Southwest Medical University, Luzhou, Sichuan Province, China.

**Keywords:** abdominal mass, case report, mesenteric cystic lymphangioma, surgical excision

## Abstract

**Rationale:**

Lymphangioma (LA) is a benign tumor that predominantly occurs in children, and is characterized by abnormal proliferation of lymphatic vessels. It is commonly present in areas such as the head, neck, and axilla. Mesenteric cystic lymphangioma (MCL) is relatively rare in adults. We reported a case of an elderly patient with a massive cystic LA of the mesentery that exerted pressure on surrounding tissues, resulting in both local and systemic symptoms.

**Patient concerns:**

The patient is a 66-year-old male who presented to our hospital with a history of recurrent abdominal pain of unknown origin for over 20 years. In the past month, the abdominal pain has worsened and is accompanied by abdominal distension.

**Diagnoses:**

Abdominal computed tomography angiography revealed a large cystic low-density mass in the right middle and lower abdomen, measuring approximately 14.9 cm × 12.8 cm × 14.9 cm. The mass contained septations, with mild enhancement of the cyst wall and septa on contrast-enhanced imaging. Small blood vessels, originating from the superior mesenteric artery, were seen traversing the lesion. Abdominal enhanced magnetic resonance imaging demonstrated a large cystic mass in the right lower abdomen, measuring approximately 17.2 cm × 14.5 cm × 8.1 cm. The mass exerted pressure on the adjacent bowel and bladder, with poorly defined borders between the lesion and the adjacent bowel.

**Interventions:**

Surgical intervention was undertaken to alleviate the patient’s symptoms and get a definitive diagnosis. The postoperative pathological results confirmed the diagnosis of cystic LA of the mesentery of the small intestine.

**Outcomes:**

The patient resumed oral intake on the third day following surgery and was discharged in stable condition on the sixth day following surgery. The patient was followed up for 1 month postoperatively without signs of recurrence.

**Lessons:**

MCL is a rare entity often presenting with nonspecific early symptoms. As the disease progresses, patients may experience abdominal pain, distension, and other gastrointestinal manifestations, potentially culminating in acute abdominal emergencies. While definitive diagnosis necessitates postoperative histopathology, preoperative imaging plays a crucial role in diagnosis. Therefore, clinicians should consider MCL in the differential diagnosis of unexplained abdominal masses.

## 1. Introduction

Lymphangioma (LA) is a benign tumor primarily composed of abnormal proliferation of lymphatic vessels. The etiology of LA remains unclear. It typically occurs in 95% of cases in the head, neck, and axillary regions, with a higher prevalence in children and relatively rare occurrences in adults.^[1]^ Mesenteric LA occurring in the abdominal cavity is exceedingly rare, accounting for <1% of all LAs, with an overall incidence estimated at approximately 1/20,000 to 1/250,000.^[2,3]^ Current case reports on mesenteric LA primarily focus on pediatric patients. This report presents the oldest patient with the longest disease history, characterized by a large tumor causing significant abdominal-pelvic mass effect and resulting in local mesenteric volvulus. The patient underwent complete surgical resection and achieved a favorable prognosis. Furthermore, this article reviews the relevant literature on small bowel mesenteric LA and introduces the following case in accordance with the CARE reporting guidelines.

## 2. Case report

The patient is a 66-year-old male who has experienced recurrent abdominal pain for over 20 years, with symptoms worsening over the past month, accompanied by abdominal distension. He has had intermittent episodes of abdominal pain for the last 2 decades, typically lasting from several minutes to hours, occasionally resolving spontaneously and sometimes requiring oral analgesics. Ten years ago, an abdominal CT scan revealed a mass near the small intestine, approximately the size of a thumb (specific imaging details unavailable). Surgical intervention was recommended, but the patient declined. Since then, his abdominal pain has recurred. In the past month, the patient’s abdominal pain has intensified, along with abdominal distension and constipation, prompting him to seek medical attention. Since the onset of symptoms, he has experienced increased urinary frequency and reduced stool output, but denies blood or melena in the stool, with no significant weight loss. The patient has a history of severe sepsis requiring hospitalization 40 years ago and has been managed for hypertension with Amlodipine besylate 5 mg daily for the past 5 years, with satisfactory blood pressure control. He denies any prior abdominal surgeries, inflammatory conditions, or exposure to toxic or radioactive substances. His personal, reproductive, and family histories are unremarkable. The physical examination revealed that the abdomen was flat and soft. A firm mass, approximately 5 cm in size, was palpable in the right lower quadrant with some mobility. There was mild tenderness in the right lower abdomen, but no rebound tenderness or muscle rigidity was noted.

**Figure 1.**
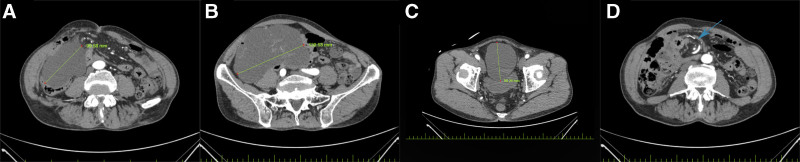
A selection of abdominal CTA images. Abdominal CTA revealed a large cystic mass in the right mid and lower abdomen, with mild enhancement of the cyst wall and septa (A–C). The arrow in the image indicates a localized mesenteric volvulus in the right mid-abdomen (D). CTA = computed tomography angiography.

**Figure 2.**
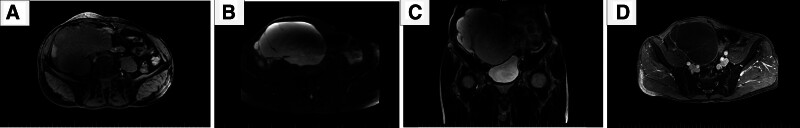
Abdominal enhanced MRI images. T1-weighted imaging (T1WI) shows slightly low signal intensity (A); T2-weighted imaging (T2WI) and SPAIR demonstrate high signal intensity (B, axial T2WI fat suppression sequence) and (C, coronal T2WI). The enhancement scan reveals significant enhancement of the cyst wall and septa (D). MRI = magnetic resonance imaging.

Initial laboratory tests revealed mild decreases in red blood cell count, hemoglobin, lymphocyte count, and lymphocyte percentage. There were slight increases in eosinophil percentage and basophil percentage. The fecal occult blood test was positive. Liver and kidney function tests, thyroid function tests, and electrolyte levels showed no significant abnormalities. Tumor markers indicated an elevated tissue polypeptide antigen, while other markers remained normal (Table 1). Abdominal computed tomography angiography (CTA) revealed a large cystic low-density mass in the right mid-lower abdomen. The mass exhibited internal septations, with mild enhancement of the cyst wall and septa on contrast imaging. Small blood vessels were observed traversing the mass, originating from the superior mesenteric artery. The borders of the mass were poorly defined, and there was compression of the adjacent bowel and bladder (Fig. 1A–C). The mesentery and mesenteric vessels in the right mid-abdomen demonstrate tortuosity, indicating malrotation of the local mesentery in this area (Fig. 1D). Abdominal enhanced magnetic resonance imaging revealed a large cystic mass in the right lower abdomen. On T1-weighted imaging, the lesion showed slightly low signal, while T2-weighted imaging and SPAIR sequences revealed high signal intensity (Fig. 2A–C). Septations were visible within the mass, with significant enhancement of the cyst wall and septa, and small vessels traversing the septa from the superior mesenteric artery (Fig. 2D). The mass had relatively well-defined borders, but there was compression of adjacent bowel and bladder, with unclear delineation from nearby bowel. Colonoscopy findings showed multiple polypoid lesions throughout the colon and rectum, the largest located at the hepatic flexure of the transverse colon, measuring approximately 0.8 cm × 1.0 cm. The remaining colonic mucosa appeared smooth, without signs of congestion, erosion, ulcers, or neoplasms.

**Table 1:** Partial laboratory test results.

Items	Results	Units	Reference range
Routine blood test			
WBC	4.85	10^9^/L	3.5–9.5
NEU	3.25	10^9^/L	1.8–6.3
MONO	0.24	10^9^/L	0.1–0.6
LYM	0.88↓	109/L	1.1–3.2
NEU-R	67	%	40–75
LYM-R	18.10↓	%	20–50
EOS-R	8.90↑	%	0.4–8
BASO-R	1.1↑	%	0–1
RBC	3.84↓	1012/L	4.3–5.8
HGB	116↓	g/L	130–175
PLT	211	10^9^/L	125–130
Biochemistry test			
ALT	14.5	U/L	9–50
AST	17.6	U/L	15–40
TBIL	11.7	μmol/L	0–23
LDH	188.1	U/L	120–150
CREA	74.8	μmol/L	57–111
UA	253.8	μmol/L	208–428
GFR	92.6	mL/L	75–145
Serum tumor markers			
TPAM	82.33↑	U/L	0.00–75.00
CEA	3.82	ng/mL	0.00–6.00
AFP	4.43	ng/mL	0.00–10.00
CA199	8.70	IU/mL	0.00–28.00
CA125	26.60	IU/mL	0.00–35.00
CA242	2.90	U/mL	0.00–10.00
CA724	5.57	IU/mL	0.00–6.00
NSE	6.14	ng/mL	0.00–15.7

AFP = alpha-fetoprotein, ALT = alanine aminotransferase, AST = aspartate aminotransferase, BASO = basophil, CA125 = carbohydrate antigen 125, CA199 = carbohydrate antigen 199, CA242 = carbohydrate antigen 242, CA724 = carbohydrate antigen 724, CEA = carcinoembryonic antigen, CREA = creatinine, EOS = eosinophil, GFR = glomerularfiltrationrate, HGB = hemoglobin, LDH = lactate dehydrogenase, LYM = lymphocyte, MONM = monocyte, NEU = neutrophil, NSE = neuron-specific enolase, PLT = platelet, RBC = red blood cell, TBIL = total bilirubin, TPAM = tumor-associated polypeptide antigen, UA = uric acid, WBC = white blood cell.

**Figure 3.**
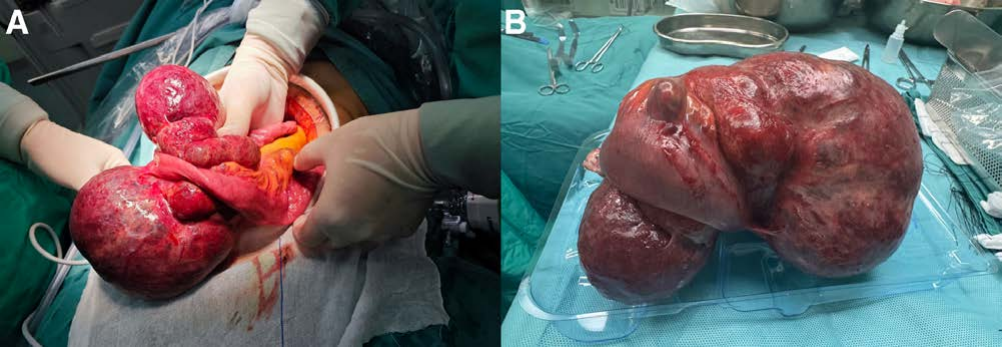
Images of intraoperative observations. The tumor appeared dark red, firm, and well-capsulated.

**Figure 4.**
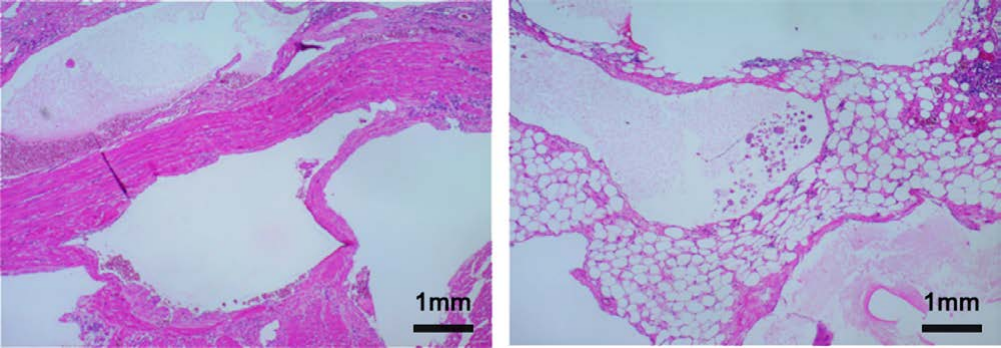
Pathological examination. (A and B) HE staining × 100 microscopic examination shows that there are multiple dilated lymphatic vessels with different sizes, and some of them are dilated into cysts.

The patient underwent laparoscopic surgery under general anesthesia. Intraoperatively, a well-capsulated tumor was found approximately 100 cm from the ligament of Treitz, measuring 20 cm × 10 cm × 10 cm. The tumor appeared dark red, firm, and well-capsulated, with clear delineation from surrounding tissues (Fig. 3). No significant hemorrhage was detected within the abdominal cavity. The liver surface showed no nodules or masses, and there were no tumor implants or metastases on the anterior wall of the stomach, gallbladder, greater omentum, colon, mesentery, or pelvic abdominal wall. Due to the tumor’s large size and severe adhesions to the small intestine, the procedure was converted to open surgery for small bowel resection and anastomosis (the excision of the small intestine tumor). Postoperative pathology revealed a 27.0 cm segment of small intestine containing a mass at the mesenteric border, measuring 17.2 cm × 11.8 cm × 6.3 cm. The mass exhibited a multilocular cystic structure, with cystic cavities ranging from 0.8 cm to 12.5 cm in diameter and wall thickness between 0.1 cm and 0.3 cm. No enlarged nodules were detected in the surrounding adipose tissue. The pathological diagnosis confirmed a mesenteric LA of the small intestine (Fig. 4). Postoperatively, the patient received a treatment regimen that included fasting, fluid replacement, and parenteral nutrition. The patient gradually resumed oral intake by postoperative day 3 and was discharged on postoperative day 6. One month post-surgery, follow-up showed no evidence of tumor recurrence.

## 3. Discussions

LA is a benign tumor characterized by the proliferation and dilation of lymphatic vessels, with its etiology and underlying mechanisms not yet fully understood. It may be associated with congenital lymphatic developmental abnormalities and impaired lymphatic drainage.^[4]^ Furthermore, studies suggest that acquired factors such as abdominal trauma, surgery, radiation, inflammation, and parasitic infections can lead to lymphatic obstruction, which is particularly linked to the development of LAs in adults, especially among the elderly.^[5,6]^ LAs occurring in the mesentery of the small intestine are extremely rare, accounting for <1% of all LAs.^[2]^ Currently, there is no reliable evidence to indicate a gender difference in the incidence of cystic LAs of the small intestine mesentery.^[7,8]^ Based on clinical and pathological characteristics, LAs are typically classified into 3 types: capillary LA, cavernous LA, and cystic LA. Notably, in cystic LA, there is no formal communication between adjacent lymphatic vessels.^[9]^ Mesenteric LAs typically manifest with nonspecific clinical symptoms and signs in the early stages. As the disease progresses, patients may experience abdominal pain, distension, palpable masses, chronic anemia, intestinal obstruction, and intussusception. Abdominal pain is the most common symptom of this condition, followed by palpable abdominal masses and gastrointestinal bleeding.^[7]^ This aligns with our case, where the patient experienced recurrent abdominal pain over an extended period. Such pain may result from external compression of the intestinal lumen, traction on the mesentery, or intestinal volvulus due to the growth of the mass.^[10–12]^ The patient’s CTA also indicated the presence of right mid-abdominal mesenteric volvulus. Some patients may present to the emergency department with acute abdominal symptoms.^[5,13]^ Typically, there is an elevation in markers of inflammation in such cases.^[5,9]^ Ultrasound demonstrates high specificity and sensitivity for cystic lesions.^[13]^ However, the diagnostic value of ultrasound for small intestine cystic LAs is limited due to interference from intestinal gas, particularly in patients presenting with acute abdominal symptoms. Computed tomography (CT) imaging is a crucial diagnostic tool for small intestine mesenteric LAs, providing detailed information on tumor density and its interactions with adjacent organs, thereby aiding in the differentiation between retroperitoneal and intraperitoneal LAs.^[3]^ Cystic LA of the small intestine typically appears on CT as unilocular or multilocular cystic lesions with homogeneous water-density contents. The cyst walls are smooth and well-defined. On contrast-enhanced CT scans, there is mild enhancement of the cyst walls and septa. Magnetic resonance imaging offers high specificity in evaluating cystic contents, facilitating precise assessment of tumors in relation to surrounding tissues.^[14]^ Cystic LAs of the small intestine typically exhibit low signal intensity on T1-weighted images and high signal intensity on T2-weighted images, often with well-defined margins.^[15]^ Endoscopic examination of the gastrointestinal tract is vital for diagnosing intraluminal LAs, as it enables lesion identification and biopsy for pathological assessment, thereby providing clear preoperative diagnosis. However, for LAs arising from the mesentery, abdominal cavity, or retroperitoneum, its diagnostic utility is relatively limited, primarily serving as an exclusionary diagnostic approach. Complete radical resection is the preferred treatment for this disease, even in asymptomatic early-stage patients. While mesenteric LAs of the small intestine are classified as benign tumors, they can be locally invasive.^[16]^ Studies show that the postoperative recurrence rate for patients undergoing complete resection is 7%, whereas for those receiving partial resection, the recurrence rate can reach as high as 50%.^[17]^ Sclerotherapy is a procedure that involves puncturing the tumor to aspirate fluid and injecting a sclerosing agent, with the goal of inducing inflammatory adhesion in the cyst wall and ultimately occluding the lymphatic vessels. Research indicates that the injection of sclerosing agents can effectively reduce the size of cysts and alleviate symptoms in children with cystic LAs of the head and neck.^[18]^ However, there is currently no reliable evidence to support the clinical efficacy of this approach for treating cystic LAs in the abdominal cavity.^[19]^ Radiofrequency ablation has demonstrated potential value in the treatment of cystic LAs in the pediatric oropharynx.^[20]^ However, its application in adult mesenteric LAs remains unexplored. Recent emerging targeted therapies for mesenteric LAs require further investigation into their therapeutic value. Mesenteric cystic LAs of the small intestine are benign tumors with an overall favorable prognosis, particularly after complete surgical resection, which is associated with a low recurrence rate. However, complications such as invasion of surrounding tissues, bowel volvulus, intussusception, bleeding, cystic hemorrhage, and infection, if not promptly and effectively managed, can lead to poor outcomes.

## 4. Conclusions

This report presents a case of a giant cystic LA of the small intestine in an elderly patient with a long history of abdominal pain. The tumor was large, causing significant mass effect, and a palpable abdominal mass was detected upon examination. Postoperative pathological analysis confirmed the diagnosis of mesenteric LA. Giant mesenteric LAs in the abdominal cavity are rare, with most patients exhibiting nonspecific clinical symptoms. Definitive diagnosis typically relies on postoperative pathological biopsy, which poses challenges for clinical practice. Therefore, it is essential for clinicians to understand common clinical presentations and be familiar with typical imaging features. Surgical intervention remains the primary treatment, and complete tumor resection is crucial for preventing recurrence.

## Acknowledgments

We extend our sincere gratitude to all individuals who contributed to this article.

## Author contributions

**Conceptualization:** Dong Xia.

**Data curation:** Wenxian Yin, Ruyi Yu.

**Formal analysis:** Wenxian Yin.

**Investigation:** Wenxian Yin.

**Writing – original draft:** Wenxian Yin.

**Writing – review & editing:** Ruyi Yu, Dong Xia.
